# Salmonella Retropharyngeal Abscess Linked to Backyard Poultry Exposure in a 12-Month-Old Girl

**DOI:** 10.7759/cureus.28375

**Published:** 2022-08-25

**Authors:** Luke Sturgill, Angela Fadil, Daniel Hinthorn, Thomas Schrepfer

**Affiliations:** 1 Department of Otolaryngology, University of Florida College of Medicine, Gainesville, USA; 2 Department of Internal Medicine, University of Kansas Medical Center, Kansas City, USA

**Keywords:** nontyphoidal salmonella (nts), deep neck space abscess, extra-intestinal salmonellosis, poultry diseases, retropharyngeal abscess

## Abstract

We report a rare complication of nontyphoidal Salmonella infection in a 12-month-old girl with a retropharyngeal abscess. The patient presented with a four-day history of nasal congestion, cough, decreased oral intake, and increased irritability. She was admitted for a suspected deep neck infection. Computed tomography confirmed a retropharyngeal abscess with airway narrowing. Incision and drainage was performed, and intraoperative cultures grew nontyphoidal Salmonella. Epidemiologic investigation revealed exposure to a backyard flock of chickens. The patient had little direct contact with chickens but did go with family to collect eggs, riding on a vehicle that likely became contaminated. This case highlights the risks to infants and young children in contact with live poultry or contaminated environments.

## Introduction

Salmonellae are ubiquitous Gram-negative bacilli responsible for up to 1.2 million cases annually in the United States. Salmonellae are broadly categorized into two groups - typhoidal and nontyphoidal [1]. Human-to-human transmission occurs in both groups. However, nontyphoidal Salmonellae (NTS) serotypes are transmitted from food and from animal sources. NTS are an important cause of gastrointestinal disease commonly associated with the consumption of poultry, eggs, and fresh produce [2]. Additional sources of infection include direct contact with reptiles or live poultry [3]. Although NTS typically produce self-limited diarrheal illness in healthy children, potential complications include bacteremia resulting in many types of extraintestinal infections [4]. In this case report, we explore a rare NTS infection in an immunocompetent 12-month-old girl with a retropharyngeal abscess (RPA) with backyard poultry as the putative source for exposure. This is the first reported case of NTS RPA linked to backyard poultry exposure in a pediatric patient.

## Case presentation

A 12-month-old immunocompetent girl was brought to the pediatric emergency department with a four-day history of nasal congestion, cough, decreased oral intake, and increased irritability. Family members denied any recent history of stridor, stertor, drooling, diarrhea, or fever. Before coming to the emergency department, she was evaluated by her pediatrician who diagnosed the patient with a viral upper respiratory infection and recommended supportive care. However, worsening symptoms prompted urgent follow-up.

Upon arrival at the emergency department, the patient was afebrile. She was noted to be irritable with a prominent muffled cry. Further physical examination findings were significant for tender left-sided cervical lymphadenopathy, left pharyngeal wall prominence, and hyperemia of the pharyngeal mucosa.

**Figure 1:**
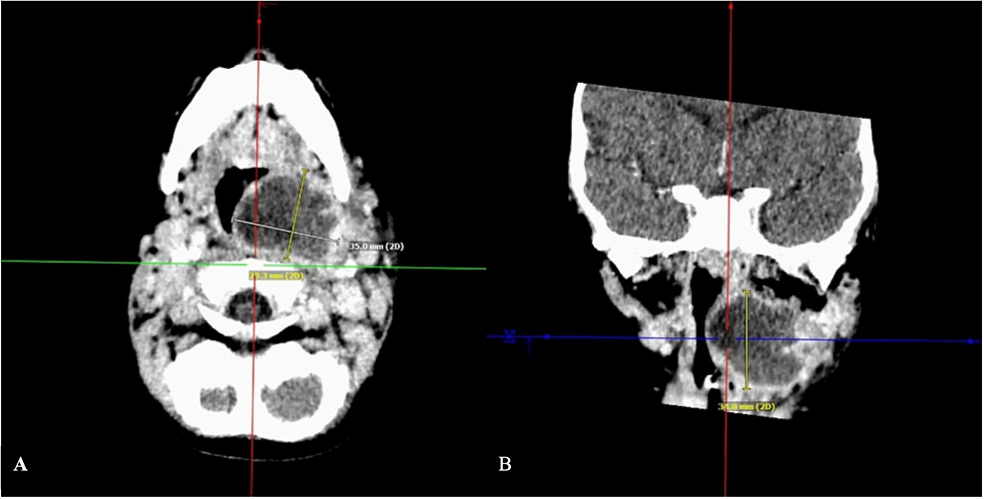
Contrasted computed tomography findings (A) axial view and (B) coronal view demonstrating a large low-attenuation fluid collection within the left para- and retropharyngeal space, resulting in airway narrowing.

In the emergency department, she was started on intravenous clindamycin 10 mg/kg every 6 h per the EMRA Antibiotic Guide and admitted for a suspected deep neck infection. Laboratory workup was significant for an elevated WBC of 23.1 × 10^9 ^cells/L and an elevated c-reactive protein (CRP) of 177.8 mg/L. A computed tomography scan of the neck with contrast showed a 3.7 × 2.8 × 3.2 cm peripherally enhancing hypodense collection with mass effect on the airway, consistent with a left retropharyngeal abscess (Figures 1A, 1B). Pediatric otolaryngology was consulted for surgical drainage, and the patient was immediately taken to the operating room. 

In the operative suite, direct laryngoscopy revealed a large bulging area over the left oropharynx that obscured direct view into the larynx. Endotracheal intubation was successful with a Miller 1 laryngoscope with grades II-III view. After the airway was secured, intraoral incision and drainage of the abscess was performed. Approximately 9 mL of purulent fluid was aspirated and sent for culture and sensitivity. After incision and drainage, a grade I view was easily obtained. The patient tolerated the procedure well and was transferred to the pediatric intermediate care unit. 

Postoperatively, the patient was continued on IV clindamycin. By the first postoperative day, she tolerated oral intake without difficulty. Through the rest of her admission, she remained afebrile without any airway concerns. On postoperative day two, she was discharged home with oral clindamycin and close follow-up. 

On postoperative day three, intraoperative cultures from the retropharyngeal abscess drainage grew nontyphoidal Salmonella. Susceptibility testing showed it to be susceptible to ceftazidime, ceftriaxone, ciprofloxacin, levofloxacin, and sulfamethoxazole/trimethoprim. Sensitivity for clindamycin was not assessed. However, given clinical improvement, clindamycin therapy was continued for seven additional days. The patient recovered uneventfully.

Retrospectively, her parents were asked about possible Salmonella exposures. The team discovered that she had regular contact with the environment of live poultry. Her grandparents raise chickens, and she joined them several times each week as they rode on a utility task vehicle (UTV) to visit the coop and harvest eggs. The patient never directly handled eggs and only occasionally entered the coop. Her grandparents recall her petting the chickens on two occasions. Her mother noted that the patient frequently touched the steering wheel and handlebars of the UTV after her grandparents handled those same surfaces with potentially contaminated hands. Otherwise, appropriate hand sanitizing precautions were taken. After each egg collection, they used hand sanitizer and washed their hands after they returned to the house.

## Discussion

RPA is a deep neck space infection that can pose immediate life-threatening complications. It predominantly occurs in children with a mean age of two to four years [5]. The retropharyngeal space, extending from the skull base to the mediastinum, contains chains of lymph nodes that are prominent in young children, but typically atrophy before puberty. Known as nodes of Rouvière, these nodes serve as a lymphatic basin for the nasopharynx and posterior paranasal sinuses [6]. Therefore, RPAs are classically present in children with upper respiratory infections that spread to these nodes. The resulting lymphadenitis can lead to cellulitis or can suppurate and progress from an organized phlegmon to mature abscess [7]. 

Children with RPA generally appear ill with moderate fever. Common findings include odynophagia, neck stiffness, and a muffled voice. As seen in this case, the prominence of the posterior pharyngeal wall can be a key physical examination finding [8]. In a stable patient without airway compromise, workup should include both lab work and neck imaging. Typical laboratory findings are elevated WBC (mean, 17,000/μL) and inflammatory markers [9]. A lateral neck plain film is valuable to assess the retropharyngeal soft tissues, which are concerning if the soft tissues near C2 are over 7 mm thick or over 14mm at C6 [10]. Proper radiographic technique is pivotal to obtaining accurate interpretation, as false thickening of the retropharyngeal space might occur if the film is captured during inspiration or if the child is crying [11]. When clinical suspicion is high or lateral neck plain film shows pathologic findings, computed tomography (CT) of the neck with intravenous contrast is the diagnostic test of choice for RPA [12]. CT also aids in differentiating RPA from cellulitis and guides planning of a surgical approach. Initial management of RPA depends on the airway status of the child and size of the abscess. In patients with severe airway compromise or large abscess, emergent surgical drainage is critical. For small abscesses (< 2.5 cm) without airway compromise, initiate a trial of empiric intravenous antibiotic therapy for 24 to 48 hours. Patients who fail to improve with conservative management are then considered for surgical drainage [13]. 

RPAs are most commonly polymicrobial. The predominant bacterial species are *Streptococcus pyogenes*, *Staphylococcus aureus*, and respiratory anaerobes including Fusobacteria, Prevotella, and Bacteroides [14]. Previously published cases describe focal Salmonella neck infections among immunocompromised adults [15,16]. However, our case is the second reported finding of a pediatric RPA due to Salmonella. The first case was reported in a 10-year-old previously healthy Taiwanese boy who developed RPA caused by Salmonella Lomita (NTS). The child’s history was negative for animal exposure but was significant for self-extraction of a molar one week prior to admission. The authors hypothesized that Salmonella infected the oral wound site and then spread through the lymphatics to the retropharyngeal space. He recovered uneventfully after surgical drainage and antibiotic therapy. Interestingly, their patient denied symptoms of gastrointestinal disease as did our patient [17]. In our case, it is plausible to assume that the patient contacted NTS from the family environment despite their attention to personal hand sanitizing. Likely, she ingested the organism due to environmental surface contamination. Although no clear mucosal breakdown was evident on examination, Salmonellae are known to penetrate the mucosal barrier in the lower gastrointestinal tract, raising questions about its pathogenicity in the upper gastrointestinal tract as well [18]. A similar case of NTS thyroid abscess was reported in a three-year-old boy with neonatal Graves’ disease after exposure to peacocks and pigeons at his grandparents’ farm [19]. However, there are no previous reports of focal RPA caused by NTS in the setting of live poultry exposure.

These cases highlight the risks to infants and young children handling live poultry or from contamination of their environment. Currently, the Centers for Disease Control and Prevention (CDC) is investigating multistate outbreaks of Salmonella infections linked to backyard poultry flocks. Approximately one-fourth of affected patients in the investigation are younger than five years old. Children and infants are particularly vulnerable, given their immature phagocytic function, propensity for poor hygiene (i.e., sticking fingers in their mouths, inconsistent hand washing), and natural curiosity. Consequently, CDC has recommended that children under five years of age should not handle chicks, ducklings, or other poultry [20].

## Conclusions

This case report calls attention to a rare manifestation of extraintestinal salmonellosis as a retropharyngeal abscess. Additionally, it demonstrates backyard poultry as a source of Salmonella infection. Although following CDC recommendations and employing good hygiene aim to reduce further outbreaks, there is a need for greater public awareness and emphasis on the infection risks associated with handling live poultry.
